# Novel velocity estimation for symmetric and asymmetric self-paced treadmill training

**DOI:** 10.1186/s12984-021-00825-3

**Published:** 2021-02-05

**Authors:** Santiago Canete, Daniel A. Jacobs

**Affiliations:** Department of Mechanical Engineering, Temple University, 1947 N. 12th Street, Philadelphia, PA 19122 USA

**Keywords:** Gait, Asymmetrya, Treadmill, Self-paced, Velocity control

## Abstract

**Background:**

Self-paced treadmills (SPT) can provide an engaging setting for gait rehabilitation by responding directly to the user’s intent to modulate the external environment and internal effort. They also can improve gait analyses by allowing scientists and clinicians to directly measure the effect of an intervention on walking velocity. Unfortunately, many common SPT algorithms are not suitable for individuals with gait impairment because they are designed for symmetric gait patterns. When the user’s gait is asymmetric due to paresis or if it contains large accelerations, the performance is diminished. Creating and validating an SPT that is suitable for asymmetric gait will improve our ability to study rehabilitation interventions in populations with gait impairment. The objective of this study was to test and validate a novel self-paced treadmill on both symmetric and asymmetric gait patterns and evaluate differences in gait kinematics, kinetics, and muscle activity between fixed-speed and self-paced treadmill walking.

**Methods:**

We collected motion capture, ground reaction force data, and muscle activity from 6 muscles in the dominant leg during walking from 8 unimpaired subjects. In the baseline condition, the subjects walked at 3 fixed-speeds normalized to their leg length as Froude numbers. We developed a novel kinematic method for increasing the accuracy of the user’s estimated walking velocity and compared our method against other published algorithms at each speed. Afterward, subjects walked on the SPT while matching their walking speed to a given target velocity using visual feedback of the treadmill speed. We evaluated the SPT by measuring steady-state error and the number of steps to reach the desired speed. We split the gait cycle into 7 phases and compared the kinematic, kinetic, and muscle activity between the fixed speed and self-paced mode in each phase. Then, we validated the performance of the SPT for asymmetric gait by having subjects walk on the SPT while wearing a locked-knee brace set to 0° on the non-dominant leg.

**Results:**

Our SPT enabled controlled walking for both symmetric and asymmetric gait patterns. Starting from rest, subjects were able to control the SPT to reach the targeted speeds using visual feedback in 13–21 steps. With the locked knee brace, subjects controlled the treadmill with substantial step length and step velocity asymmetry. One subject was able to execute a step-to gait and halt the treadmill on heel-strikes with the braced leg. Our kinematic correction for step-length outperformed the competing algorithms by significantly reducing the velocity estimation error at the tested velocities. The joint kinematics, joint torques, and muscle activity were generally similar between fixed-speed and self-paced walking. Statistically significant differences were found in 5 of 63 tests for joint kinematics, 2 of 63 tests for joint torques, and 9 of 126 tests for muscle activity. The differences that were statistically significant were not found across all speeds and were generally small enough to be of limited clinical relevance.

**Conclusions:**

We present a validated method for implementing a self-paced treadmill for asymmetric and symmetric gaits. As a result of the increased accuracy of our estimation algorithm, our SPT produced controlled walking without including a position feedback controller, thereby reducing the influence of the controller on measurements of the user’s true walking speed. Our method relies only on a kinematic correction to step length and step time which can support transfer to systems outside of the laboratory for symmetric and asymmetric gaits in clinical populations.

## Background

Treadmill training can potentially improve the health-related quality of life in populations with gait impairments, such as stroke [1–3], spinal cord injury [4, 5], lower-limb amputation [6, 7], Parkinson’s disease [8], and multiple sclerosis [8, 9]. In addition to improvements in physical function, increasing physical activity can also improve socio-emotional outcomes (e.g. reducing depression, improving participation, and sense of well-being [10, 11]).

In a gait laboratory, treadmill training can both facilitate exercise and provide a robust environment for assessing the effect of rehabilitation interventions. Standard gait analysis during fixed-speed walking can provide effective measurements of joint kinematics, joint torques, and metabolic energy expenditure. In contrast, clinically relevant measurements, such as changes in self-selected walking speed, are challenging to measure on a fixed-speed treadmill. One method for measuring self-selected walking speed in a gait laboratory is by utilizing a self-paced treadmill (SPT) [12–20]. On an SPT, the velocity is controlled in real-time based on the kinematic and kinetic measurements of the user. The instantaneous measurements of the SPT can provide clear measurements of how a user’s self selected walking speed can change due to interventions such as assistive devices, sensory modulation, and biofeedback.

Training on an SPT may provide additional benefits over fixed speed treadmill walking. High-intensity, task-oriented, random practice leads to larger changes in cortical plasticity during training [21–23]. SPTs provide an environment where the users can both freely select the intensity of the task and experience variability in velocity that is directly related to their gait performance. Increasing the variability of the training routine, especially through increased demands in accuracy and amplitude of the motor task, may improve standard treadmill training [24, 25]. SPTs may be more closely related to overground walking than fixed-speed treadmills because the user’s gait performance on an SPT directly leads to changes in belt velocity compared to the changes in relative position that would occur on a fixed-speed treadmill. Furthermore, the instantaneous walking velocity measurements on an SPT can be incorporated into biofeedback methods, increasing motivation by showing training targets or overall improvement through the course of a training session [26].

Previous estimation algorithms can be grouped according to the information used in the controller. The most common estimation algorithms are: (1) using a position feedback controller to drive a set of markers on the pelvis or torso to the center of the treadmill [12–16], (2) estimating changes in the center of mass velocity by integrating anterior-posterior ground reaction force data [17, 18], and (3) estimating velocity based on approximate relationships between kinematic variables, such as leg-swing velocity and torso velocity [19, 20].

Algorithms for SPT training have improved over the past two decades, but there are still major limitations restricting applicability to rehabilitation. Two key limitations of existing SPT algorithms are: (1) they perform poorly in asymmetric gaits, making them unsuitable for many individuals with gait impairments, and (2) they cannot fully separate the measurement of the user’s desired walking velocity from the transient response of the controller. Both of these limitations are the result of poor velocity estimation at each step. For this reason, many kinematic and force-based methods include an additional position feedback controller or attempt to smooth velocities between steps to overcome the error in the estimation [18, 19, 27].

It is clear that the performance of an effective SPT is based on the accuracy of the estimate of the user’s velocity. Not only does feedback control not solve the fundamental problem of velocity estimation but it also introduces substantial error in the measurement of the user’s velocity. When feedback control or smoothing is employed to reduce error, it establishes a relationship whereby the current treadmill velocity is based on information from previous steps. This creates clear issues for individuals with gait asymmetry, where assuming smoothness of left and right steps is invalid. The controller misidentifies a real change in velocity as an error and introduces a transient signal (i.e. it modifies treadmill velocity and acceleration) on future steps that leads to inaccurate measurements in velocity. Even in the case of symmetric gaits, large single-step accelerations and deceleration will also be misidentified as position errors and produce large transients. To achieve safe self-pacing for individuals with gait asymmetry (e.g. velocity, step length, and step time), the SPT must be able to maintain performance through the alternating periods of acceleration and deceleration between each step without relying on feedback systems that do not have guaranteed stability nor any guaranteed bounds on the accumulation of error.

We present a novel method for a self-paced treadmill that overcomes the above limitations and enables controlled walking for asymmetric gait and large changes in accelerations. We introduce a kinematic correction to step-length that has increased accuracy in estimating the user’s walking velocity. Our SPT controller takes the estimated velocity directly and updates the treadmill’s velocity at each heel strike. In contrast to previous studies, our SPT produces controlled self-pacing without feedback control or smoothing, thereby eliminating the transient velocity signals that interfere with the measurement of the user’s true velocity. Our underlying control method is kinematic-based and relies only on step time and step-length, which has the potential to be transferred to systems outside the laboratory.

In addition to enabling self-pacing, our velocity estimation can also be used to improve measurements of step length during standard fixed-paced treadmill walking. Historically, researchers have chosen kinematic and kinetic estimation methods to overcome challenges in measuring walking velocity using step length on the treadmill. Overground, heel position can be marked exactly with gait mats, or even simple contact marking [28], permitting accurate step and stride length measurements. However during treadmill walking, the treadmill belt is a moving reference frame and therefore the user’s overall gait velocity is a function of both the belt velocity and the subject’s velocity relative to the treadmill belt. Any relative movement of the user on the treadmill during the step directly changes the distance between the markers at heel strike, making the measurement ineffective at non-steady velocities [29].

The purpose of the present study was to validate a self-paced treadmill system for enabling symmetric and asymmetric gaits and to compare kinematics, kinetics, and muscle activity between fixed-speed and treadmill walking. Our goal was to answer three questions: (1) Could we improve the accuracy of the velocity estimation with respect to previous methods in order to avoid the use of control and smoothing functions? (2) Can unimpaired subjects walk on the self-paced treadmill at a target speed when given visual feedback? (3) Can unimpaired subjects walk comfortably on the treadmill with asymmetry induced via a locked knee brace? We hypothesized that our kinematic method would significantly reduce the error in velocity estimation, permitting control of the SPT without a continuous feedback loop. Secondly, we hypothesized that subjects would be able to successfully start and maintain a stable velocity using visual feedback. Lastly, we hypothesized that there would be minor differences in kinematics, kinetics, and muscle activity between fixed-speed and self-paced modes.

## Methods

### Data collection

We recruited 8 subjects with no history of neurological or musculoskeletal impairment, and no prior experience walking on a self-paced treadmill. The subjects in this study were: 2 Females and 6 Males, (mean ± std) Age $23.75 \pm 3.79$ years, Mass $72.00\pm 10.36$ kg, Foot Length $0.27\pm 0.01$ m, Leg Length $0.92 \pm 0.06$ m. All subjects were right-dominant. We tracked the motion of the subjects using 16 motion capture cameras (sample rate: 120 Hz; Qualisys, Goteborg, Sweden) and 39 reflective markers (34 lower body, 5 upper body). We measured the ground reaction forces using a split-belt instrumented treadmill (sample rate: 1200 Hz; Bertec, Ohio, USA). We collected surface electromyography (EMG) from muscles in the dominant leg (i.e soleus, tibialis anterior, lateral gastrocnemius, biceps femoris long head, rectus femoris, and vastus lateralis) using a wired amplifier system (Delsys, Massachusetts, USA).

### Experimental protocol

The experiment consisted of three separate trials performed in the following order: (1) fixed-speed treadmill (FST) walking, (2) self-paced treadmill (SPT) walking at a target velocity, and (3) self-paced treadmill walking with induced asymmetry. For the FST trial, subjects walked at three velocities, normalized to their leg length so that they correspond to the three Froude Numbers of 0.075, 0.15, and 0.225 [30, 31]. We calculated leg length as the average height of the left and right greater trochanter markers during the static trial and calculated foot length as the distance from the toe marker to the calcaneus marker on the shoe.

For the SPT walking at a target velocity trial, we instructed subjects to match the target velocities displayed on a large television screen placed in front of the treadmill. We used the default GUI Control for the treadmill, which displayed velocities for the left and right belt with a precision of 2 decimal places. We repeated the trials 3 times, once for each of the Froude numbers we used in the fixed-speed trial.

For the SPT walking with induced asymmetry trial, the subjects donned a locking knee support brace that was set to 0° of flexion. We instructed the subjects to walk on the SPT with two different levels of asymmetry: (1) walk as normally as possible, and (2) walk as asymmetrically as possible.

All subjects were given a few minutes to acclimate to the system only before the first trial of the induced asymmetry and target velocity tests. Before the acclimation period, we informed subjects to take a step to start the treadmill, and to stop walking while keeping both feet on the ground to halt, or in case of an emergency to lift both feet off the ground to halt. The acclimation period ended when the subjects indicated they were ready to proceed. In all self-paced trials the subjects started from rest and the treadmill initiated when they took the first step. At the end of the trials, we asked the subjects to stop walking to halt the treadmill.

### Self-paced treadmill algorithm

#### Gait velocity estimation

**Fig. 1:**
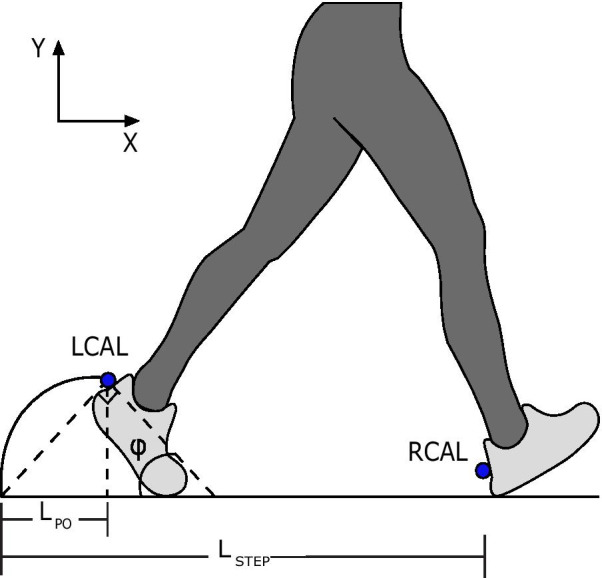
Illustration of trailing leg push-off. On a treadmill, the absolute location of the calcaneus markers are a function of both belt velocity and the relative velocity between the user and the belt. At faster walking velocities, ankle motion during push-off shorten the estimated distance between the calcaneus markers ($\mathrm {LCAL}$ and $\mathrm {RCAL}$). We improved the velocity estimation error by calculating the push-off length ($\mathrm {L}_{PO}$) and adding it to the difference in the calcaneous markers to find total step length ($\mathrm {L}_{step}$).

**Fig. 2:**
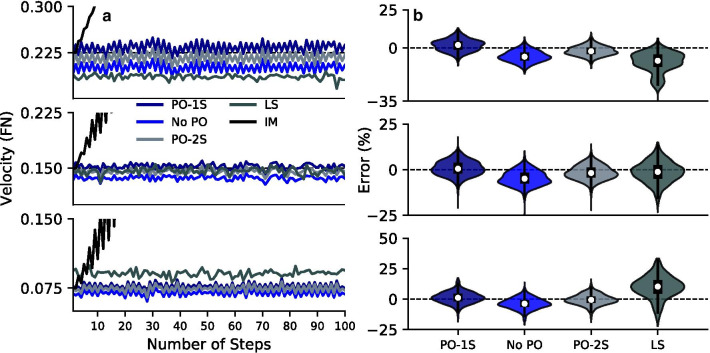
Velocity estimation error during fixed-speed treadmill walking for the three tested velocities. **a** Mean estimated velocities across subjects for several kinematic and kinetic algorithms using 100 steps on the treadmill in fixed-speed mode. **b** Distribution plots of the velocity estimation error for each of the algorithms. The impulse-momentum algorithm is not shown because it accumulates significant error. Using the 1-segment push-off correction to step length (PO-1S) resulted in a statistically significant reduction in the velocity estimation error at all three normalized velocities with respect to the no push-off model (No PO) (Signed-Rank Test: Froude Number = 0.075, p = 0.0016; Froude Number = 0.150, p = 0.0078; Froude Number = 0.225, p = 0.0078). We did not observe any significant differences between the 1-segment model (PO-1S) and the 2-segment model (PO-2S). The 1-segment model (PO-1S) resulted in a statistically significant reduction of error at the lowest and fastest speeds with respect to the leg-swing method (LS) (Signed-Rank Test: Froude Number = 0.075, p = 0.0078; Froude Number = 0.150, p = 0.0547; Froude Number = 0.225, p = 0.0156)

In our implementation, we made the simplifying assumption that the segments of the foot behave as a single rigid body in which the trailing foot makes a right triangle with the hypotenuse along the ground (Fig. 1). In our preliminary testing, we evaluated a version with a two-segment foot model but found no significant change in the estimation accuracy (Fig. 2). We calculate the new step length and step-time at each heel-strike. Below, we list the calculation for a right step, which is performed at the moment of right heel-strike.

The angle of the trailing foot ($\phi$) was calculated at the instant of heel-strike of the leading foot. Assuming the trailing foot and the ground form a right triangle pivoting about the tip of the toes we can calculate the angle ($\phi$) as:

1 $$\begin{aligned} \phi = \arcsin \left( \dfrac{\mathrm {LCAL_{y}}}{L_{foot}}\right) \end{aligned}$$

At the instant of heel-strike of the leading leg (right), we calculate a push-off correction ($L_{PO}$) that estimates the anteroposterior distance traveled by the calcaneus marker ($\mathrm {LCAL}_{x}$) if the foot was flat on the ground before the rotation of the ankle. The correction is a function of the angle ($\phi$) and the *y* position of the CAL marker ($\mathrm {LCAL}_{y}$)

2 $$\begin{aligned} L_{PO} = \mathrm {LCAL}_{y} \tan (\phi ) \end{aligned}$$

We measured the difference in the anteroposterior position between the CAL markers of the leading and trailing feet at the instant of heel-strike of the leading foot. The total step length is the sum of the push-off distance ($L_{PO}$) and the difference in calcaneal markers. If the swinging foot contacts the ground behind the stance foot (i.e. in a step-to gait), the step length is set to zero.

3 $$\begin{aligned} L_{step} = {\left\{ \begin{array}{ll} 0, \qquad \mathrm {RCAL}_{x} \le \mathrm {LCAL}_{x}\\ \mathrm {RCAL}_{x} - \mathrm {LCAL}_{x} + L_{PO}, &{} \text {otherwise} \end{array}\right. } \end{aligned}$$

In a similar way, we calculated the difference in time between the instant of heel-strike of the trailing foot and the instant of heel-strike of the leading foot.

4 $$\begin{aligned} t_{step} = t_{RHS} - t_{LHS} \end{aligned}$$

Finally, we obtained the walking velocity as the distance traveled over the time duration for each step.

5 $$\begin{aligned} V_w = \frac{L_{step}}{t_{step}} \end{aligned}$$

#### Control implementation

Our system consisted of three nodes: (1) the motion capture system sent the marker and force data, (2) the treadmill sent out current velocity and received velocity commands, and (3) the velocity algorithm computed and sent the treadmill velocity command based on the received marker and force data. We wrote custom Python 2.7 code using the equipment’s API to create the nodes and implemented the communication between nodes using the Robot Operating System (ROS) [32].

To detect heel strikes, we filtered the ground reaction forces using a 3rd order, digital infinite impulse response (IIR) filter with 50 Hz cutoff and calculated the time when the filtered vertical force value rose above 5% of the subject’s body weight [33] measured during the static trial. We selected the filtered order and bandwidth based on our preliminary data. Since the filter implementation is single pass, there is a fixed delay. We found that this was the highest order filter we could use while keeping the delay under the period of a camera capture frame (1/120 s). For these filter parameters, the delay in filtered force was 6 ms. We did not implement any phase shift correction after filtering and calculated the marker position and stance time directly based on the filtered force data.

We used a two-value calculation for belt acceleration. For most steps, the acceleration of the treadmill belt was capped at 2.0 m/s$^2$. However, we also defined a small velocity change as a single-step difference of 0.3 m/s. When the single-step difference was less than that value, as occurs during fast symmetric walking, we set the belt acceleration to be 7.0 m/s$^2$. Because the treadmill velocity command occurs at heel-strike, the user has already accelerated their torso before the treadmill velocity changes. As a result, the person will move forward on the belts before the belts get to the commanded velocity. By increasing the acceleration, we can minimize the delay and reduce the amount of movement of the person relative to the ground frame. All stop commands were set with an acceleration equal in magnitude to 1/4th of the current speed. These values were selected experimentally and if needed they can be easily modified to match the subjects comfort level.

In addition to the velocity estimation, we implemented four optional features for safety and control. These features can be turned off at will depending upon the study:

1. *Halt if flight detected* The treadmill is stopped if both feet are off the ground for 1 frame of motion capture data (120 Hz). This will stop the users if they transition from walking to running. The user can also halt the treadmill simply and safely by making a short hop.
2. *Halt if long double stance detected* During preliminary testing, we calculated double stance time for multiple velocities and fitted an exponential curve to the data as a function of belt velocity. The exponential curve was truncated to 0.25 and 0.60 s at the extremes. If the stance time was greater than the fitted stance time, we assumed the subject has decelerated in a single step and we stopped the treadmill.
3. *Ignore step if crossover detected* If we detected a step that exceeded the double stance threshold time, we determined if any of the foot markers (e.g. calcaneus, 1st metatarsophalangeal joint, or great toe), crossed the center line of the belt. If a crossover occurred, we discarded the step and maintained the last commanded velocity.
4. *Virtual wall correction* Rarely, a subject can accelerate or decelerate so much in a single step, they can approach the extremes of the treadmill. If the calcaneus markers came within 150 mm of the back end of the treadmill, the next commanded velocity was reduced by 20%. If the toe markers came within 175 mm of the front of the treadmill, the next commanded velocity was increased by 10%. Each modification of the velocity applied only to the next velocity command immediately after the virtual wall event. No modification occurred for any steps after the single step correction.

### Data and statistical analysis

The content and timing of a set of ROS messages can be recorded using the ROS framework bag system. The advantage of this system is that researchers can accurately replicate an experiment and fairly compare performance between algorithms. In order to evaluate changes between the velocity estimation with and without the push-off correction, and to evaluate changes between the proposed algorithm, the impulse-momentum method, and the leg-swing method, we extracted the data from the bags recorded during the fixed-speed trials.

#### Algorithm comparison

For the fixed-speed trials, we trimmed 100 total steps counting from the end of the trial and evaluated the RMS sample error between the velocity estimates and the treadmill belt speed for each subject. To compare the algorithms, we implemented only the velocity estimation aspect of two algorithms used in the literature: (1) Leg Swing Velocity as described by Yoon et. al. [19], and (2) Impulse-Momentum through the integration of the anterior-posterior ground reaction forces [17, 18]. In addition, we also compared our model to the velocity estimate with no push-off correction, and to a push-off model using 2 segments.

#### Self-paced treadmill—target velocities

For the SPT with target velocity trial, we calculated the error between the treadmill belt speed and the target speed using the first 100 steps of the trial once the subjects reached steady state walking. We determined the number of steps it took to reach steady walking when the walking velocity was within a 90% range of the target speed. We also measured the pelvis position of the subjects throughout the trials.

#### Self-paced treadmill—asymmetric gait

For the asymmetry trial, we used 100 steps from the end of the trial and split them into left-right and right-left groups. We calculated the ratios of step length, step time, and step velocity as braced leg divided by non-braced leg (B/NB). We also measured the pelvis position of the subjects throughout the trials.

#### Joint kinematics and kinetics

For the kinematic and kinetic comparison between the fixed and self-paced trials, we took 100 steps for each subject and each condition. The force data was filtered using a fourth-order zero-lag low-pass Butterworth (15 Hz). The joint kinematics and kinetics were obtained using the OpenSim 4.0 API [34]. The resulting joint angles and torques were normalized in time expressed as a percentage of the gait cycle. Then, the joint torques were normalized in magnitude to each subject’s mass. We split the angles and torques in 7 phases of the percent gait cycle (0–12, 12–30, 30–50, 50–62, 62–75, 75–87, 87–100), and calculated the average angles and torques for each percent gait phase within every step for the three tested velocities.

#### Muscle excitations

For the EMG comparison between the fixed and self-paced trials, we took 100 steps for each subject and each condition. The EMG signals were full-wave rectified, filtered using a fourth-order zero-lag passband Butterworth filter (10-500Hz), and smoothed with a fourth-order zero-lag low-pass Butterworth filter (15Hz). The EMG signals were normalized in time expressed as a percentage of the gait cycle. Then, normalized in magnitude expressed as a percentage of the peak EMG of the ensemble average for each muscle across all trials [35]. We split the normalized signals into 7 phases of the percent gait cycle (0–12, 12–30, 30–50, 50–62, 62–75, 75–87, 87–100) and calculated the average EMG for each percent gait phase within every step for the three tested velocities.

#### Statistical analyses

All statistical tests were implemented using Matlab 2018b. We first evaluated the normality of the data using the Anderson-Darling test. The test rejected the null hypothesis that the data belonged to a normal distribution for all comparisons. Then, we tested the self-paced and fixed-speed populations to check if they had equivalent distributions using the Jarque-Bera test. If we failed to reject the null hypothesis, then we tested for differences between the self-paced and fixed-speed conditions using the Wilcoxon signed rank test. Otherwise, we tested using the sign test. All statistical tests were set to a significance level of 0.05.

## Results

### Velocity estimation error of different algorithms

We found that for all three velocities, the RMS error of 100 steps for each subject was significantly less when the 1-segment push-off (PO-1S) correction was applied compared to the no push-off (No PO). For the three tested velocities, the mean percentages in which the correction increased the measured step length with respect to No PO were (mean ± std: Froude Number = 0.075, 5.10% ± 3.17%, Froude Number = 0.15, 5.67% ± 2.34%, Froude Number = 0.225, 7.84% ± 4.37%). The PO-1S model compared to the 2-segment push-off model (PO-2S) had no significant differences in RMS error at any of the three velocities. The PO-1S algorithm had a statistically significant reduction in RMS error compared to the leg swing (LS) velocity algorithm at the lowest and fastest speeds (FN: 0.075, FN: 0.225). The impulse-momentum algorithm was left out of the statistical comparison since it resulted in an accumulation of error that is out of the scale of the other three methods (Fig. 2). The median RMS estimation errors for the PO-1S were 4.87%, 3.66%, and 3.67%, while the LS were 11.59%, 5.40%, and 9.65% for the three tested velocities respectively (Table 1).

**Table 1:** Across subject median (uncorrected p-value) of the RMS percentage velocity error in the estimated velocity of 100 steps for each subject during fixed-speed treadmill walking

Froude number	Push-off (1S)	No push-off	Push-off (2S)	Leg swing
0.075	4.87	5.53 (0.0156)*	4.48 (0.0703)	11.59 (0.0078)*
0.150	3.66	6.23 (0.0078)*	3.72 (0.7266)	5.40 (0.0547)
0.225	3.67	5.96 (0.0078)*	3.20 (1.0000)	9.65 (0.0156)*

* Statistically significant difference between the listed group and the One-Segment(1S) pushoff model at 0.05 significance. Legend: FN - Froude Number, 1S—One-Segment Foot Model, 2S— Two-Segment Foot Model

### Self-paced treadmill target velocity control

**Fig. 3:**
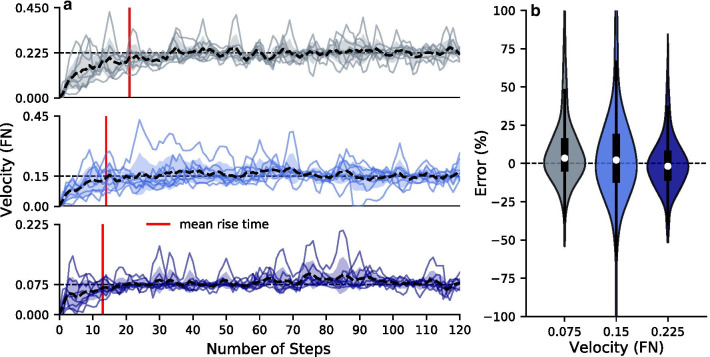
Target velocity error during self-paced trials for the three tested velocities. **a** Walking velocity (normalized to Froude number) for 8 subjects at the three target velocities. The average number of steps taken to reach a steady walking speed were: 13, 14, and 21 at the three speeds respectively. **b** Distribution plots of the percentage error between target and subject walking velocities calculated in standard units (m/s). Across subjects, the inter-quartile range of the error included zero for the three tested velocities

**Fig. 4:**
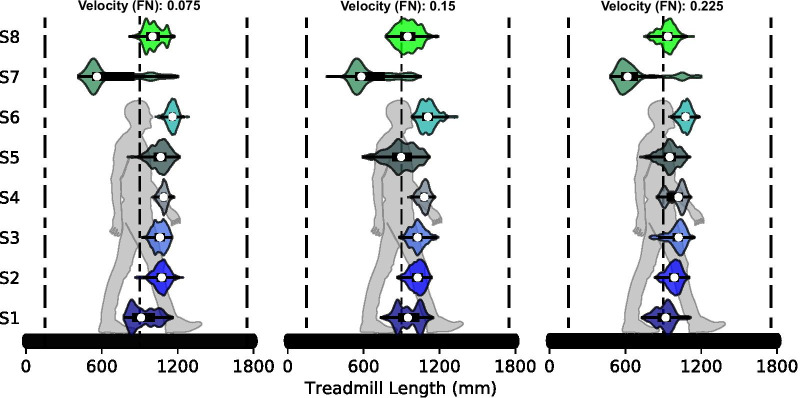
Pelvis position during self-paced trials. All subjects were able to maintain their position within a 1 m range on the treadmill without the need of a position control loop showing that the velocity estimation is stable without position feedback. One subject (S7) shifted their location during the first few steps for the three velocities, and walked closer to the back of the treadmill for the rest of the trial. The front and back vertical dashed lines represent the location of the virtual walls in the safety protocol. The front wall was not reached by any subject during the three trials, and the back wall was reached an average of 0.55, 0.87, and 0.75 times per 100 steps for the three speeds respectively

All of the subjects were able to control the treadmill to the three target velocities. The average number of steps to achieve a steady speed were 13, 14, and 21 at the three tested speeds respectively. The majority of the subjects were able to keep zero error within the inter-quartile range of the 100 sample steps (Fig. 3). The median errors for the 800 steps sampled were 3.48%, 2.26%, and − 1.80% for the three target velocities respectively. The front virtual wall was not reached by any subject, and the back virtual wall was reached an average of 0.55, 0.87, and 0.75 times per 100 steps for the three target velocities respectively (Fig. 4). Before the first target velocity trial, subjects took $3.57\,\pm \,1.27$ minutes to feel comfortable with the device.

### Self-paced treadmill asymmetric gait control

**Fig. 5:**
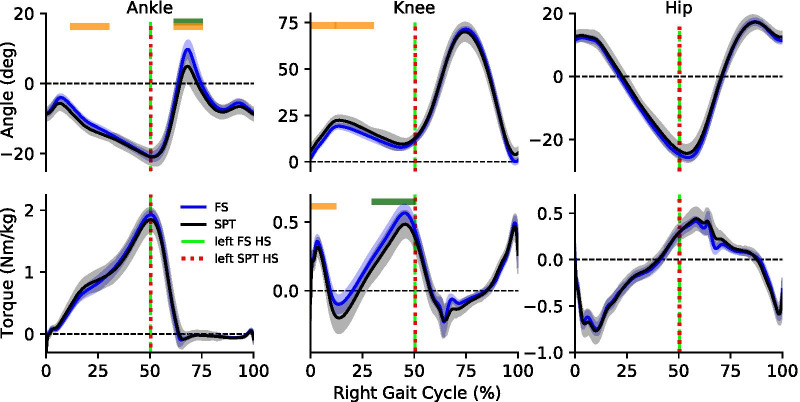
Average joint angles and torques for the intermediate speed (FN: 0.15). In general, the joint angle and joint torque trajectories for the lower leg were consistent between fixed and self-paced trials. The dashed vertical lines show the instant of contralateral heel strike which was consistent between fixed and self-paced conditions. We observed some statistically significant differences in the ankle and knee, especially at the middle speed. Each block represents one of the seven selected phases of the gait cycle. The horizontal blocks (FN: 0.075, dark red; FN: 0.15, orange; FN: 0.225, dark green) represent statistical significance $(\text {P}<0.05)$ for the corresponding gait phase

With the knee brace on, subjects were able to control the self-paced treadmill at both levels of asymmetry. For the first level, the medians across subjects for step length (SL), step time (ST) and step velocity (SV) ratios between the braced and non-braced legs (B/NB) were: SL—1.03, ST—1.14 and SV—0.90. For the second level the median ratios were: SL—0.41, ST—1.13 and SV—0.37 (Fig. 5a). The front virtual wall was not reached by any subject, and the back virtual wall was reached an average of 3.43, and 1.29 times per 100 steps for the two levels of asymmetry respectively (Fig. 5b). In the brace only trial, one novice subject chose to quickly circumduct the braced leg to walk faster on that side. When instructed to exaggerate the asymmetry in the second trial, a more adept subject adopted a step-to gait and completely stopped the treadmill by having the braced leg land behind the opposite leg at heel-strike, thus producing a negative step length. Before the first asymmetry level trial, subjects took 5.23 ± 2.13 minutes to feel comfortable with the device.

### Kinematic and kinetic differences

**Fig. 6:**
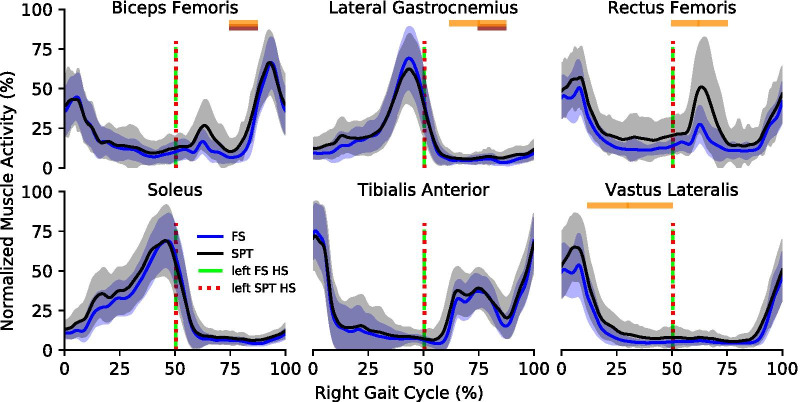
Average EMG activity for the Intermediate Speed (FN: 0.15). In general, the normalized muscle activity was consistent between the measured muscles. The dashed vertical lines show the instant of contralateral heel strike which was consistent between fixed and self-paced conditions. We observed some statistically significant differences in the extensor and flexor muscles around the knee. Each block represents one of the seven selected phases of the gait cycle. The horizontal blocks (FN: 0.075, dark red; FN: 0.15, orange; FN: 0.225, dark green) represent statistical significance $(\hbox {P}<0.05)$ for the corresponding gait phase

At the slowest speed, we observed no significant differences between fixed-speed and self-paced conditions in joint angles and torques. At the middle speed, we observed a significant difference in ankle angle (median difference: 1.60°) during middle stance and initial swing (median difference: 2.13°), for the knee angle during initial loading (median difference: − 2.96°) and middle stance (median difference: 2.65°), and for the knee torque (median difference: − 0.052 Nm/kg) during initial loading. At the fastest speed, we observed a significant difference in ankle angle (median difference: 2.72°) during initial swing, and knee torque (median difference: − 0.048 Nm/kg) during late stance. (Table 2). The heel-strike of the contralateral foot for the self-paced and fixed-speed conditions happened at 50% of the gait cycle (Fig. 6).

**Table 2:** Across subject median(uncorrected p-value) of the mean difference (SPT–FS) in the joint angles and normalized torques of 100 steps for each subject

Muscle	Froude number	Initial loading	Mid stance	Late stance	Pre. swing	Initial swing	Mid. swing	Late swing
		(0–12%)	(12–30%)	(30–50%)	(50–62%)	(62–75%)	(75–87%)	(87–100%)
Ankle angle	0.075	–	–	–	–	–	–	–
	0.150	–	− 1.60(0.0078)	–	–	− 2.13(0.0078)	–	–
	0.225	–	–	–	–	− 2.72(0.0078)	–	–
Knee angle	0.075	–	–	–	–	–	–	–
	0.150	2.96(0.0078)	2.65(0.0078)	–	–	–	–	–
	0.225	–	–	–	–	–	–	–
Hip angle	All Speeds	–	–	–	–	–	–	–
Ankle torque	All Speeds	–	–	–	–	–	–	–
Knee torque	0.075	–	–	–	–	–	–	–
	0.150	− 0.052(0.0078)	–	–	–	–	–	–
	0.225	–	–	− 0.048(0.0078)	–	–	–	–
Hip torque	All Speeds	–	–	–	–	–	–	–

* Statistically significant difference between fixed-speed and self-paced treadmill walking at 0.05 significance

### Muscle excitation differences

**Fig. 7:**
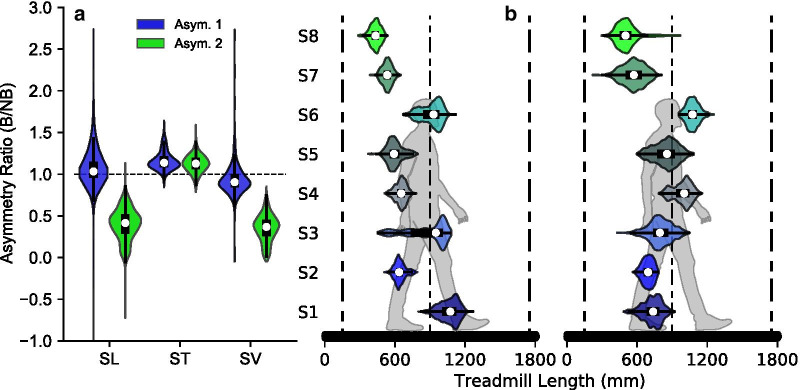
Gait asymmetry measurements and pelvis position on the self-paced treadmill. *a* The unimpaired subjects were able to maintain consistent velocity walking with induced asymmetry via a locked knee brace at 0° flexion. When given instructions to produce more asymmetry, in the second level, subjects chose to increase the asymmetry of step length rather than step time. The zero values of step length and step velocity show that subjects walked with a “step-to” gait. SL—step length, ST—step time, SV—step velocity. *b* All subjects were able to stay within a 1 meter range on the treadmill at both levels of asymmetry without the need of a position control loop. The front wall was not reached by any subject during the two trials, and the back wall was reached an average of 3.49, and 1.29 times per 100 steps for the two levels of asymmetry respectively. The front and back vertical dashed lines represent the location of the virtual walls

At the slowest speed, we observed a significant difference between fixed-speed and self-paced conditions in EMG activity for the biceps femoris (median difference: 5.37%), and lateral gastrocnemius (median difference: 0.57%) during middle swing. At the middle speed, we observed significant differences in the biceps femoris during middle swing (median difference: 3.34%), for the lateral gastrocnemius during initial swing (median difference: 0.93%) and middle swing (median difference: 1.72%), for the rectus femoris during preswing (median difference: 7.90%) and initial swing (median difference: 12.91%), and for the vastus lateralis during middle stance (median difference: 4.42%) and late stance (median difference: 1.41%). At the fastest speed, we observed no significant differences for all muscles (Table 3). The heel-strike of the contralateral foot for the self-paced and fixed-speed conditions happened at 50% of the gait cycle (Fig. 7).

**Table 3:** Across subject median(uncorrected p-value) of the mean difference (SPT–FS) of the normalized muscle activity in 100 steps for each subject

Muscle	Froude number	Initial loading	Mid stance	Late stance	Pre. swing	Initial swing	Mid. swing	Late swing
		(0–12%)	(12–30%)	(30–50%)	(50––62%)	(62–75%)	(75–87%)	(87–100%)
Bic. Fem.	0.075	–	–	–	–	–	5.37(0.0078)*	–
	0.150	–	–	–	–	–	3.34(0.0078)*	–
	0.225	–	–	–	–	–	–	–
Lat. Gas.	0.075	–	–	–	–	–	0.57(0.0078)*	–
	0.150	–	–	–	–	0.93(0.0078)*	1.72(0.0078)*	–
	0.225	–	–	–	–	–	–	–
Rect. Fem.	0.075	–	–	–	–	–	–	–
	0.150	–	–	–	7.90(0.0078)*	12.91(0.0078)*	–	–
	0.225	–	–	–	–	–	–	–
Soleus	All Speeds	–	–	–	–	–	–	–
Tib. Ant.	All Speeds	–	–	–	–	–	–	–
Vas. Lat.	0.075	–	–	–	–	–	–	–
	0.150	–	4.42(0.0078)*	1.41(0.0078)*	–	–	–	–
	0.225	–	–	–	–	–	–	–

*Statistically significant difference between fixed-speed and self-paced treadmill walking at 0.05 significance

## Discussion

### Comparison of different methods for estimating walking velocity

Accurate velocity measurements require precise measurements of both step length and step time. In general, measuring step time is more accurate than measuring step length on a treadmill because the sampling frequency of the treadmill is higher than the sampling frequency of the motion capture system. Estimating step length from foot markers is challenging, especially when there are large changes in acceleration in the belt speed or in the user’s velocity relative to the belt. Because the belt is moving relative to the fixed camera coordinate system, and the user moves relative to the belt, the location of the foot markers can be variable. However, the relative distance between markers at the instant of heel-strike can be measured reliably. At common walking speeds, the ankle has plantar-flexed and the heel has lifted off the ground at the moment the leading leg contacts the ground. Humans rely on step lengthening to increase walking speed [36]. To achieve these faster speeds, the angle between the legs at each step increases and this additional extension causes increased plantar flexion in roll-off in the trailing leg.

We found that incorporating the ankle roll-off into the estimate of step length produced more accurate velocity estimations during treadmill walking. With our proposed algorithm, the median error of the velocity estimation for the 8 subjects was between − 0.90 and − 1.40% (Fig. 2). Because there is greater roll-off as step length increases, the correction makes a greater contribution to the total step length as the subject walks faster. Given that step time can be measured accurately, the negative median value suggests that the push-off correction produces slightly greater step lengths than the true value.

Our primary motivation for using a single segment foot model was to reduce the number of markers required to track the foot during the push-off period, especially in real-time situations where the loss of a marker might result in a lost-step calculation. For validation, we compared the accuracy of the 1-segment model to a 2-segment model. The results showed no statistically significant difference in the estimated velocity.

The proposed algorithm had significantly greater accuracy than the velocity estimation based on the integration of anterior-posterior forces in the impulse-momentum method. It is well known that the integration of noisy signals leads to accumulation of error, even when filtered appropriately. The value of the integrated error must be reset periodically for the signal to be relevant. It is unclear when this should be done for force-based methods. Accelerations in the belt also reduce the accuracy of the force measurements on the treadmill due to frictional forces and belt inertia. Kim et al., labelled the changes in anterior-posterior force as an “anomalous force” and described a method for reducing the unwanted forces in order to increase similarities between treadmill and overground walking [37]. Hsiao et. al. showed that the positive portion of the anterior-posterior impulse during walking in individuals post-stroke is a poor predictor of self-selected walking velocity ($R^2$ of 0.34) and cannot explain changes in walking velocity ($R^2$ of 0.01) [38]. The accuracy of the calculation of the anterior-posterior impulse also degrades with changes in velocity [37]. One reason is that the impulse-momentum requires a previous state to update the velocity estimate which leads to an accumulation of error [27]. As a result, impulse-momentum algorithms also include a position control loop [18], in order to reject periods where acceleration occurs over more than a single step, or rely on an additional external tethered force sensor that can further inhibit the user’s intended movement [17].

The leg-swing algorithm resulted in a significant increase in RMS error with respect to the proposed algorithm. Our results are in accordance with Yoon et. al. and suggest that the assumption of the foot swing velocity being sinusoidal does not behave well even for an unimpaired population. To overcome the inaccuracy the authors interpolated the walking speed of the subject to adjust the velocity estimates. One limitation of the interpolation is that it is done combining the two feet which in our eyes assumes symmetric walking. Another limitation is that the interpolation is extrapolated from fixed-speed to self-paced walking (i.e. we can expect greater variability in self-paced walking), which are not equivalent at least in swing time [19].

It is unclear how the assumptions in the leg-swing method would hold for an impaired population, especially for certain types of gait impairments where a subject would circumduct or perform a step-to gait. During our asymmetry tests, one subject performed a step-to gait making the treadmill stop and move as they walked. Although this subject did not progress with one leg, they did swing, which would result in a non-zero velocity command via the leg-swing method. So, it is clear that a subject could swing their leg without progressing forward which can only be compensated by incorporating a controller of some sort. Lastly, it is unknown if updating the treadmill speed in middle swing is beneficial, especially for subjects with impaired balance.

Position control algorithms have well-established issues with velocity estimation because the position data must be differentiated, which reduces the signal to noise ratio. Filtering this signal can introduce biases. Several research groups have proposed different velocity estimators (e.g. Luenberger observer, Kalman Filter) to overcome the accuracy problems. Unfortunately, these methods do not behave well in response to natural variability in velocity or for the user’s desire for large accelerations or deceleration. Large transients in walking velocity have lead to large errors in the control loop [15]. As a result, position control loops interfere with the ability of the researcher to collect accurate data and can lead to erroneous interpretations of gait interventions when measured on a self-paced treadmill. Furthermore, there have been key studies that show that humans may use velocity-control rather than position-control when walking on a treadmill [39]. In this case, interpreting a change in position as a desired change in velocity is erroneous.

### Self-paced treadmill target velocity control

All subjects were able to safely control the average velocity of the treadmill with visual feedback. The average error of all subjects and velocities was 1.17%, which is approximately 0.01 m/s for the study velocities. The visual GUI gives velocity feedback with a minimum resolution of 0.01 m/s as well. Thus, subjects on average had excellent control of the treadmill. All subjects were able to take steps at the target velocity and most were able to keep the target velocity within their interquartile range. All subjects were able to maintain their pelvis position within a 1 meter range throughout the trial (Fig. 4) without the need of a position control loop.

Subject 6 triggered a stop due to a period of long double stance during the trial but then was able to rapidly bring the treadmill back up to the target velocity and maintain it for the rest of the trial (Fig. 3). Thus, the variability in velocities in Fig. 3 comes from actual variation in user’s speed and not from measurement noise. In general, the median error was closer to zero for the higher velocities. However, this is potentially due to practice effects since velocities were presented in increasing order.

We found that by increasing the acceleration to 7 m/s$^2$ we could reduce the positional shift safely during steady walking. To eliminate the chance of inducing slip, we created a gain scheduling system allowing us to drop the acceleration to a safe level for any large change in belt speed. For most steps (especially at asymmetric walking) the treadmill acceleration was 2.0 m/s$^2$, only when the walking velocity of the subject was steady (less than 0.3 m/sec difference from step to step) the acceleration was set to 7 m/s$^2$. These values can be easily tuned for any level of impairment in any future system replicating our method. We found these values to be comfortable for all tested subjects including asymmetric walking.

The front virtual wall was not reached in any of the three self-paced trials and the back virtual wall was rarely reached. Although, the instances per 100 steps at the back wall were rare (0.55%, 0.87%, 0.75%) it may be a consequence of the slight overestimate in velocity of the proposed algorithm. Other potential reasons to reach either wall could be: (1) the subject crossed over and the velocity was not updated, (2) the subject took a very long step reaching the front of the treadmill before the new step was recorded, (3) the subject took a very short step in a long period of time reaching the back of the treadmill before the new step was recorded. The distinction between our virtual wall and position control is important. Position control is a feedback control method which is active at every instant in time. Our virtual wall is a finite step correction that is designed as a safety feature for large single-step changes in velocity.

### Self-paced treadmill asymmetric gait control

Subjects were able to safely start, stop, and maintain a self-selected velocity on the treadmill while walking with induced asymmetry due to the knee brace. In response to the locked knee brace, subjects chose a strategy of increasing step length asymmetry to maintain forward velocity. Step time asymmetry was preserved across levels. All subjects were able to maintain their pelvis position within a 1 m range throughout the trial (Fig. 5b) without the need of a position control loop.

At the highest level of asymmetry, our subjects achieved ratios of step length, step time, and step velocity asymmetry that are comparable to published data on a post-stroke population [40–42]. Although, research on impaired groups has shown higher temporal asymmetry than what we measured during our study, step time is an accurate measurement and we surmise that changing step time and increasing temporal asymmetry will not reduce the accuracy nor robustness of our proposed algorithm. Overall, our system was able to tolerate substantial step to step differences in step length, time, and velocity, and control the treadmill velocity accordingly. Therefore, we believe our system could serve as an adequate training environment in the future for clinical populations, such as stroke.

### Kinematics and kinetic differences

While the temporal joint angle and torque profiles for fixed-speed and self-paced conditions were similar, we observed some statistically significant differences. Although there were enough steps to determine statistical significance, the differences between conditions are relatively small and may not rise to the level of clinical relevance. The absolute value of the median differences in joint angles were less than 3°. The absolute value of the differences in joint torques were near 0.05 Nm/kg. Furthermore, the changes in walking pattern were not consistent across velocities, which also suggests that the statistically significant differences are not clinically relevant. Our results agree with previous studies where there was large similarity between kinematics and kinetics between fixed and self-paced walking [16].

### Muscle excitation differences

Similarly to the kinematic and kinetic comparison, while the temporal EMG activity profiles for fixed-speed and self-paced conditions were similar, we observed some statistically significant differences. Most differences did not happen consistently through all velocities. We do not believe the differences observed in lateral gastrocnemius and vastus lateralis to be clinically relevant. The major difference happened in the rectus femoris during preswing and initial swing at the middle speed. One possible reason can be that subjects walked with higher speed variability at the middle speed (Fig. 3), resulting in higher rectus femoris EMG activity for those that walked above the target velocity [43].

### Limitations

One limitation of the results on joint kinematics, joint kinetics, and muscle activity is that we chose to do fixed paced walking before the self-paced walking for all subjects to acclimate them to the treadmill before the target velocity control and asymmetry trials. Although subjects were given the option to rest and the periods of walking were short (4–6 min), there is the possibility that some of the differences are related to accumulated fatigue.

One current limitation of the proposed algorithm is that discrete steps are needed. If a subject drags one limb and the ground reaction force does not drop below the 5% threshold, this algorithm would not detect a step.

Another limitation that arises from kinematic algorithms is that they are dependant on marker data, especially in real-time situations where the loss of a marker might result in a lost-step calculation. For this reason, we preferred a simpler one segment model to reduce the number of markers required to track the foot during the push-off period. One of the next steps we will take will be to implement an additional method for estimating the push-off angle in case of a lost CAL marker, potentially using an alternative marker.

For this study, we used an instrumented treadmill and a motion capture system. Such equipment is expensive and requires dedicated spaces which could be seen as a limiting factor in a clinical setting. However, the proposed algorithm is not tied to these systems, and similar measurements could be obtained through insole pressure sensors and inertial measurement units. As a matter of fact, one limitation of the current setup is the need for the subject to step on the corresponding belt for accurate step detection which could be overcome with the use of insole sensors.

## Conclusion

We have proposed a novel self-paced treadmill system for accurately measuring the user’s self-selected walking velocity on a treadmill. Our system robustly measures gait velocity when users walk with both symmetric and asymmetric spatiotemporal patterns without the use of smoothing or position control loops that add transient noise to the measurements. By eliminating spurious measurements of the self-paced treadmill control from the user’s self-selected velocity, we can improve the ability of researchers and clinicians to analyze gait performance and test interventions on the treadmill for individuals with gait impairment.

## Abbreviations

API: Application programming interface
B: Braced
EMG: Electromyographic or electromyography
FN: Froude number
FST: Fixed-speed treadmill
GUI: Graphical user interface
IIR: Infinite impulse response
RCAL: Right calcaneus
RHS: Right heel-strike
RMS: Root mean square
ROS: Robot operating system
LCAL: Left calcaneus
LHS: Left heel-strike
NB: Not braced
PO: Push-off
SL: Step length
S: Subject
SPT: Self-paced treadmill
ST: Step time
SV: Step velocity
